# Structural Insights into Phylloquinone (Vitamin K1), Menaquinone (MK4, MK7), and Menadione (Vitamin K3) Binding to VKORC1

**DOI:** 10.3390/nu11010067

**Published:** 2019-01-01

**Authors:** Nolan Chatron, Abdessalem Hammed, Etienne Benoît, Virginie Lattard

**Affiliations:** USC 1233 RS2GP, INRA, VetAgro Sup, Univ Lyon, F-69280 Marcy l’Etoile, France; nolan.chatron@vetagro-sup.fr (N.C.); abdessalem.hammed@vetagro-sup.fr (A.H.); etienne.benoit@vetagro-sup.fr (E.B.)

**Keywords:** vitamins K, phylloquinone, menaquinones, menadione, VKORC1, membrane, structural interactions, molecular modeling, enzymatic assays

## Abstract

Vitamin K family molecules—phylloquinone (K1), menaquinone (K2), and menadione (K3)—act as γ-glutamyl carboxylase (GGCX)-exclusive cofactors in their hydroquinone state, activating proteins of main importance for blood coagulation in the liver and for arterial calcification prevention and energy metabolism in extrahepatic tissues. Once GGCX is activated, vitamin K is found in the epoxide state, which is then recycled to quinone and hydroquinone states by vitamin K epoxide reductase (VKORC1). Nevertheless, little information is available concerning vitamin K1, K2, or K3 tissue distribution and preferential interactions towards VKORC1. Here we present a molecular modeling study of vitamin K1, menaquinones 4, 7 (MK4, MK7), and K3 structural interactions with VKORC1. VKORC1 was shown to tightly bind vitamins K1 and MK4 in the epoxide and quinone states, but not in the hydroquinone state; five VKORC1 residues were identified as crucial for vitamin K stabilization, and two other ones were essential for hydrogen bond formation. However, vitamin MK7 revealed shaky binding towards VKORC1, induced by hydrophobic tail interactions with the membrane. Vitamin K3 exhibited the lowest affinity with VKORC1 because of the absence of a hydrophobic tail, preventing structural stabilization by the enzyme. Enzymatic activity towards vitamins K1, MK4, MK7, and K3 was also evaluated by in vitro assays, validating our in silico predictions: VKORC1 presented equivalent activities towards vitamins K1 and MK4, but much lower activity with respect to vitamin MK7, and no activity towards vitamin K3. Our results revealed VKORC1’s ability to recycle both phylloquinone and some menaquinones, and also highlighted the importance of vitamin K’s hydrophobic tail size and membrane interactions.

## 1. Introduction

Vitamin K is of main importance for many physiological processes as the exclusive cofactor of the γ-glutamyl carboxylase (GGCX) enzyme, which catalyzes a post-translational modification: γ-glutamyl carboxylation. This leads to the activation of vitamin K-dependent proteins (VKDPs) involved in bone metabolism [1,2], cancer progression and inflammatory response [3], oxidative stress [4], sphingolipid synthesis [5], and pancreas exocrine activity [6]. Vitamin K is also crucial for blood coagulation [7] (clotting factors are hepatic VKDPs), and for energy metabolism and arterial calcification prevention through the activation of osteocalcin [8] and matrix Gla protein [9], respectively, both being extrahepatic VKDPs. The GGCX enzyme is only activated by vitamin K in its hydroquinone state (vitamin K^H^), after which vitamin K^H^ is converted to vitamin K epoxide (vitamin K^E^), showing no biological activity [10].

Biological reduction of vitamin K^E^ to vitamin K quinone (vitamin K^Q^) was evidenced in 1970 [11], and the gene coding for the enzyme catalyzing this reduction was discovered in 2004 [12,13]. The 163-amino acid protein named vitamin K epoxide reductase (VKORC1) has been shown to support vitamin K^E^ to vitamin K^Q^ reduction, and then vitamin K^Q^ to vitamin K^H^ reduction [14,15]—no activity was evidenced towards vitamin K^H^. VKORC1 presents four strictly conserved cysteine residues among all species: C43, C51, C132, and C135 [16,17]. C132 and C135 residues form the catalytic site of VKORC1 (the CxxC motif being the active site of most oxidoreductases family proteins), whereas C43 and C51 were shown to contribute to redox transfer to this catalytic site [18,19,20]. An enzymatic mechanism based on biochemistry studies was suggested for vitamin K reduction by VKORC1 in the 1980s and still remains valid: the sulfur atom of a cysteine residue (C135 for VKORC1) is responsible for a nucleophilic attack targeting the carbon 2 atom of vitamin K^E^ [21].

Vitamin K is a family of molecules composed of phylloquinone (vitamin K1), menaquinones (vitamin K2), and menadione (vitamin K3), and little information about their respective participation in biological processes is available in the literature [22]. Vitamin K tissue distribution is heterogenous, with vitamin K1 mainly present in the liver, heart, and pancreas. Vitamin K2 is found in high levels in the brain and kidney [23]. Vitamin K1 has been proved to be the main form used as GGCX cofactor, but vitamin K2 can also perform this function [24], and both vitamins K1 and K2 support the blood coagulation mechanism [25]. Nevertheless, vitamin K2 studies revealed biological activity when its hydrophobic tail is composed of two to six isoprene units, but this activity decreased when hydrophobic tail size increased [26]. Vitamin K3 (showing no hydrophobic tail) has been proved to be inactive towards coagulation [27,28] and constitutes vitamins K1 and K2 catabolite product [29]. Nonetheless, the potential interactions of vitamins K1, K2, and K3 with VKORC1 remain uncharacterized, with previous studies mainly focused on vitamin K1 in its epoxide state [30,31,32]. In the present work, we aimed at describing VKORC1 activity towards different forms and states of vitamin K, to define whether the enzyme reduces preferential substrates. We thus investigated binding of vitamins K1, MK4, MK7, and K3 in their epoxide, quinone, and hydroquinone states, through molecular modeling studies; then we evaluated enzymatic activity in the presence of vitamins K1, MK4, MK7, and K3 in the epoxide state.

Despite the remaining controversy about VKORC1 3D structure—some publications suggested a three-transmembrane helix (3 TM) structure [33,34,35,36], whereas some others show proof of a 4-TM structure [19,37,38,39]—recent publications have described experimental and modeling evidence of a 4-TM structure [30,31,39]. Moreover, one study indicated the C51–C132 disulfide bridge conformation of VKORC1 as primary [30]. Our previously developed 3D model fitted well with these data. It has been shown to correctly predict affinity and rank the efficiency of four VKORC1 inhibitors—warfarin, acenocoumarol, phenprocoumon, and difenacoum—using binding free energy (BFE) calculation in good correlation with experimentally measured inhibition constants. It also evidenced the C51–C132 disulfide bridge conformation as the only one able to bind vitamin K1^E^ [32], so we used it to perform molecular docking and molecular dynamics (MD) simulations.

The present work generated an overall characterization of vitamin K–VKORC1 interactions. We expected to obtain an overview of vitamin K–VKORC1 interactions, giving us structural insights into VKORC1’s ability or inability to reduce some vitamin K forms and to induce VKOR activity into the liver and extrahepatic tissues, with respect to their vitamin K status. *In silico* and *in vitro* investigations were highly consistent in explaining VKORC1’s ability to support specific vitamin K reduction, and may constitute a great base for the future development of therapies, considering the different forms of vitamin K to use.

## 2. Materials and Methods 

### 2.1. Molecular Modelling, Docking, and Dynamics of Vitamin K and VKORC1 Human Protein

Vitamin K 3D structures were downloaded from ChemSpider website (www.chemspider.com). The VKORC1 model was used and the molecular dynamics (MD) system was built and prepared as in our previous paper [32]. Molecular docking of vitamin K with respect to VKORC1 was carried out using AutoDock 4.2 software [40]; 100 runs of molecular docking were carried out for each type of vitamin K, using the Lamarckian Genetic Algorithm. Molecular dynamics (MD) simulations were performed using GROMACS 5.1.2 package software [41], and the CHARMM-36 force field was applied [42]. Each vitamin K–VKORC1 complex was previously embedded into DLPC membrane [43] using the *membed* function of GROMACS software. Solvation, energy minimization, 100-ps temperature equilibration, and 1-ns pressure equilibration were carried out using GROMACS routines. 100-ns MD simulations were performed in replica on each vitamin K–VKORC1 complex, and then the two trajectories were concatenated and analyzed as one 200-ns MD simulation.

### 2.2. MD Analysis and Figures

Distance measurements between the vitamin K carbon-2 atom and C135 α-carbon atom in MD simulations were performed using the *dist* function of GROMACS. Binding free energy (BFE) on vitamin K–VKORC1 or vitamin K–membrane complexes and the VKORC1 residue contributions to BFE were determined using the Molecular Mechanics Poisson–Boltzmann Surface Area (MM-PBSA) method [44] implemented for GROMACS software [45]. Graphics were drawn using Grace software [46] and 3D figures were prepared using PyMOL software [47].

### 2.3. VKOR Activity Assays and Kinetics

**Figure A1:**
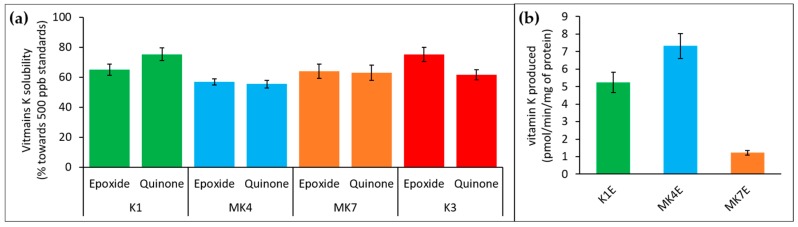
Vitamin K epoxide and quinone solubility in assay conditions and vitamin K epoxide reductase (VKOR) activity assays and kinetics towards 10-µM vitamins K^E^ solutions. (**a**) Vitamin K1, MK4, MK7, and K3 epoxide and quinone solubility in 100-µM buffer solutions. Areas under the curve obtained from LC-MS standards were normalized for each vitamin K epoxide and quinone (100%) and used as references to evaluate solubility of vitamins K in our buffer solution. Solubility values are colored as green, blue, orange, and red for vitamins K1, MK4, MK7, and K3, respectively. Resulting solubility values ranked within the same range of values, from 55% for vitamin MK4^Q^ to 75% for vitamin K1^Q^, and thus assessed the equivalent concentrations of vitamin K epoxide in our buffer solutions. (**b**) Specific vitamin K epoxide reductase activity towards vitamins K1^E^ or MK4^E^. VKOR activity was evaluated in the presence of 10 µM of vitamins K^E^.

Recombinant human VKORC1 was expressed in membranes of *Pichia pastoris* as described previously [48]. Microsomal VKOR activity and liquid chromatography - atmospheric pressure chemical ionization tandem mass spectrometry (LC-APCI/MS/MS) analyses were assayed as described in our previous paper [49] with some modifications, especially concerning vitamin K3 analysis. Stock solutions of vitamin K epoxide at 50 mM were prepared in Triton (vitamins K1, MK4, and K3) or n-hexane (vitamin MK7). Standard reactions were performed in 200 mM of 4-(2-hydroxyethyl)-1-piperazineethanesulfonic acid (HEPES) buffer (pH 7.4) containing 150 mM KCl, 1 mM dithiotreitol, and 1.5 g/L of total proteins. Here, 20 µL of vitamin K epoxide (K1, MK4, MK7, or K3) solution in 1% Triton X-100 at various concentrations (i.e., 5 mM or 0.5 mM) were added to initiate the reaction. After incubation at 37 °C for 30 min, the reaction was stopped by addition of 2 mL of isopropanol (vitamins K1, MK4, and MK7) or methanol (vitamin K3). Protein precipitation was then performed by centrifugation at 3000 g for 10 min. For vitamin K3, methanol layer was removed and directly dried under nitrogen. For vitamins K1, MK4, and MK7, supernatant was collected and 2 mL of hexane were added. After 3000 g centrifugation for 10 min, the hexane layer was removed and dried under nitrogen. The dried residue was immediately dissolved in 200 µL of methanol, and the reaction product was finally analyzed by liquid chromatography-mass spectrometry (LC-MS). With respect to vitamin K3, the mobile phase was composed of 45% methanol (instead of 96% for vitamins K1, MK4, and MK7), 55% water, and 1/1000 formic acid. Then, 173.1 and 189.0 ions were monitored, corresponding to vitamins K3^Q^ and K3^E^, respectively. VKOR activity was assessed by performing the same assay adding 1.56 µM warfarin, leading to 40% inhibition of this activity. Vitamin K epoxide and vitamin k quinone solubilities in assay conditions were controlled (Figure A1) to ensure VKORC1 enzymatic activity comparison towards vitamin K1, MK4, MK7, and K3.

## 3. Results

**Figure A2:**
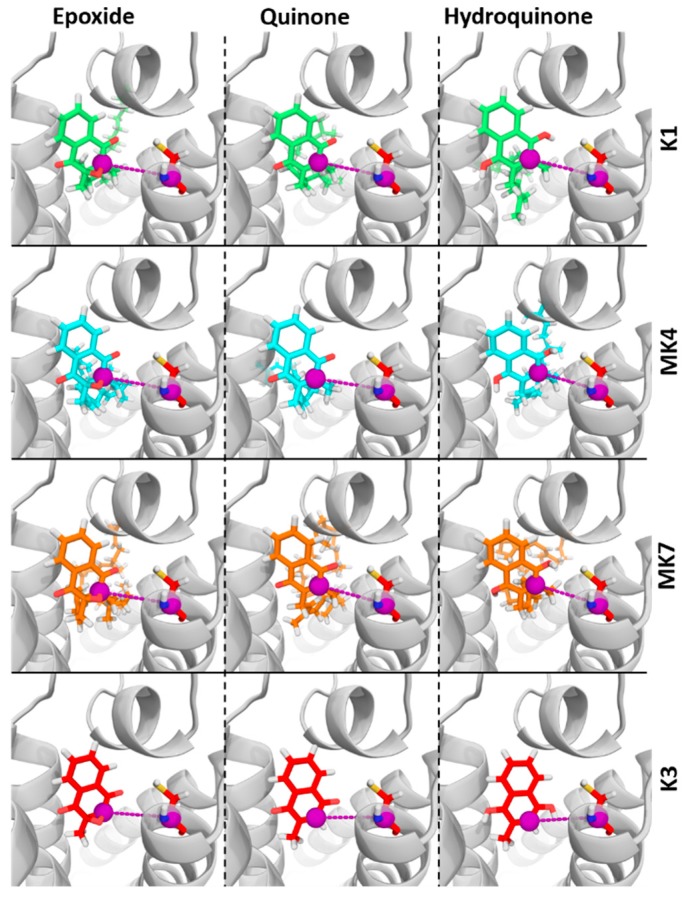
Molecular docking of vitamins K to vitamin K epoxide reductase (VKORC1) and distance measurement between vitamins K and C135 residue. VKORC1 3D structure is shown as a transparent grey image and the C135 residue as red sticks. Vitamin K is shown as sticks and colored in green, cyan, orange and red for K1, MK4, MK7, and K3, respectively. Oxygen, nitrogen, sulfur and hydrogen atoms are colored in red, blue, yellow, and white, respectively. The carbon-2 atom of vitamin K and the C135 α-carbon atom are shown as violet spheres, and the distance measured in molecular dynamis (MD) simulations is depicted as violet dashes.

Vitamins K1, MK4, MK7, and K3 in their epoxide, quinone, and hydroquinone states were docked on our previously developed VKORC1 structural model [32]. All vitamins K bound the same VKORC1 site, close to C135 residue, with the naphthoquinone head facing transmembrane helices (TMs) 1 and 4 (Figure A2). Then, 100-ns molecular dynamics (MD) simulations were performed in replica on each vitamin K–VKORC1 complex, and finally analyzed.

### 3.1. Calculated Distance between Vitamin K and the VKORC1 Catalytic Site in MD Simulations

**Figure 1:**
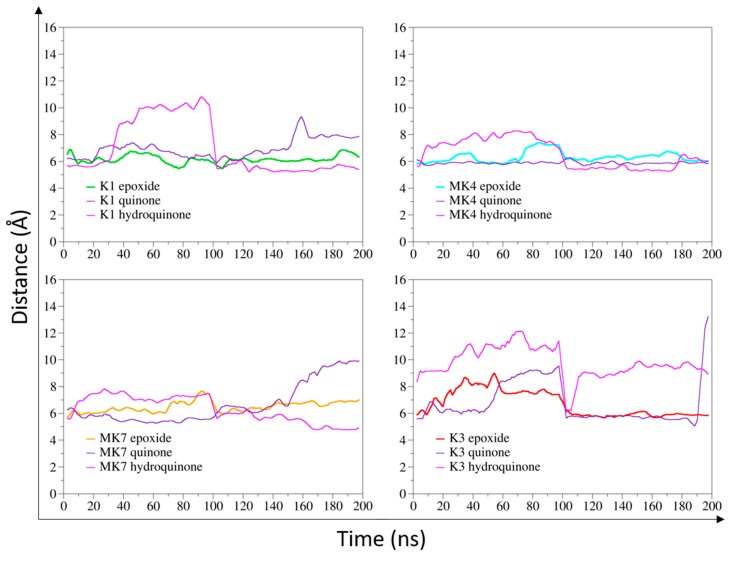
Distance measurement between the carbon-2 atom of vitamin K (K1, MK4, MK7 and K3 in their epoxide, quinone, and hydroquinone states) and the vitamin K epoxide reductase (VKORC1) catalytic site (α-carbon of the C135 residue) in molecular dynamics (MD) simulations. The epoxide states of vitamins K1, MK4, MK7, and K3 are shown as green, cyan, orange, and red lines, respectively. The quinone state is depicted as a violet line and hydroquinone as a pink line, for each form of vitamin K. Two 100-ns MD simulations were performed for each vitamin K–VKORC1 complex, then concatenated to be considered as one 200-ns MD simulation.

The distances between the carbon-2 atom of vitamin K and the α-carbon atom of C135 residue (Figure A2) were measured in MD simulations (Figure 1). The α-carbon of C135 was considered instead of the sulfur atom to avoid fluctuations induced by the C135 sidechain motion.

Vitamin K1^E^, as well as vitamins MK4^E^, MK4^Q^, and MK7^E^ were close to C135, since distance values remained between 6 and 8 Å in the 200-ns MD simulations. Vitamins K1^Q^ and MK7^Q^ were close to the catalytic residue for 150 ns; then the distance reached higher values than 8 Å. Distance between the hydroquinone state of vitamins K1, MK4, and MK7 and C135 residue were around or higher than 8 Å for at least 100 ns in each MD simulation. The distance between vitamin K3 and C135 constantly reached values greater than 8 Å, regardless of the epoxide, quinone, or hydroquinone state.

### 3.2. Structural Characterization of Vitamin K Binding to VKORC1 

Interactions of two biomolecules forming a complex can be described using the binding free energy (BFE), which can be likened to the biological affinity between those molecules. The BFE is always a negative value, and corresponds to the energy that one would have to apply to the complex to separate the two biomolecules. The lower the BFE is, the higher the affinity is. BFE calculation was then applied to our complexes (formed by VKORC1 and vitamins K1, MK4, MK7, and K3 in epoxide, quinone, and hydroquinone states) in MD simulations (Table 1).

#### 3.2.1. Overall Binding Free Energy of Vitamin K–VKORC1 Complexes

BFE calculations revealed heterogenous values depending on the different forms and states of vitamin K. Complexes formed by VKORC1 and vitamins K1^E^, K1^Q^, and MK4 (regardless of the reduction state) had very close values, from −51.49 to −55.91 kcal/mol, with standard deviations from 3.35 to 4.49 kcal/mol. Nonetheless, the affinity of vitamin K1^H^ towards VKORC1 was lower: the calculated BFE for this complex was −48.48 kcal/mol. Moreover, the high value of standard deviation (7.79 kcal/mol) revealed instability of vitamin K1^H^ binding to VKORC1 in MD simulations. Vitamin MK7 affinity towards VKORC1 seemed to be higher, as shown by the BFE values that reached −61.67 and −57.42 kcal/mol for vitamins MK7^E^ and MK7^H^, respectively. However, vitamin MK7^Q^ had a BFE similar to vitamin K1^H^, with a value of −47.96 kcal/mol. The most striking observation was the high values of standard deviations concerning all states of vitamin MK7 (from 7.24 to 9.95 kcal/mol) showing great instability of vitamin MK7 binding to VKORC1. Finally, vitamin K3 presented the weakest affinity towards VKORC1, with BFE values from −20.19 to −22.19 kcal/mol.

Contribution of VKORC1 amino acids to vitamins K binding was then investigated to carry on this vitamin K–VKORC1 interaction characterization.

#### 3.2.2. The VKORC1 Amino Acid Contribution to Vitamin K Binding

**Figure 2:**
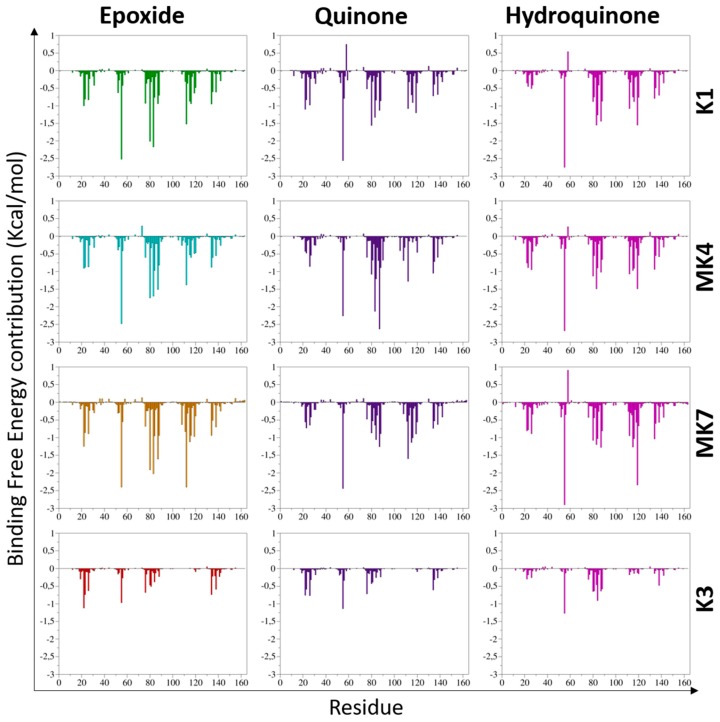
Contribution of vitamin K epoxide reductase (VKORC1) amino acids to the binding of vitamins K1, MK4, MK7, and K3 in their epoxide, quinone, and hydroquinone states. VKORC1 residue contributions to vitamins K binding are shown as green, cyan, orange, and red bars for the epoxide state of vitamins K1, MK4, MK7, and K3, respectively. VKORC1 residue contributions to vitamin K binding are presented as violet and pink bars for the quinone and hydroquinone states, respectively. Two 100-ns molecular dynamics (MD) simulations were performed on each vitamin K–VKORC1 complex, then concatenated to be considered as one 200-ns MD simulation.

The calculated BFEs have been decomposed into a single amino acid contribution, allowing us to identify VKORC1 residues of main importance in the overall affinity towards vitamins K (Figure 2). Residues showing BFE contribution lower than −1 kcal/mol were considered as crucial for vitamin K binding.

The residue F55 was identified as greatest-contributing amino acid to the calculated BFE in each vitamin K–VKORC1 complex. N80, F83, and F87 (carried by TM2) and L112 (located on TM3) residues were highly involved in vitamin K1, MK4, and MK7 binding, whereas TM4 residues A115 and G116 only participated in vitamin MK7 binding. Finally, L22 residue (TM1) contributed in the epoxide state of vitamins K1, MK4, and MK7 binding, whereas G84 (TM2) and Y119 (TM3) residues were involved in their quinone and hydroquinone state binding, respectively. 

VKORC1 residues appeared as poorly contributing to vitamin K3 binding, which was consistent considering the weak affinity revealed by BFE calculated for vitamin K3–VKORC1 complexes.

Structural investigations were then carried out to study crucial amino acids spatial location and to describe their effect on vitamins K binding and orientation towards VKORC1 catalytic site.

#### 3.2.3. Vitamins K–VKORC1 Binding Overview

Amino acids contribution to the previously calculated BFE was shown on the VKORC1 3D structure, leading us to determine their structural relationship towards vitamin K. The presence of hydrogen bonds was also investigated to produce an accurate and overall characterization of vitamin K–VKORC1 interactions.

##### Vitamin K1

**Figure 3:**
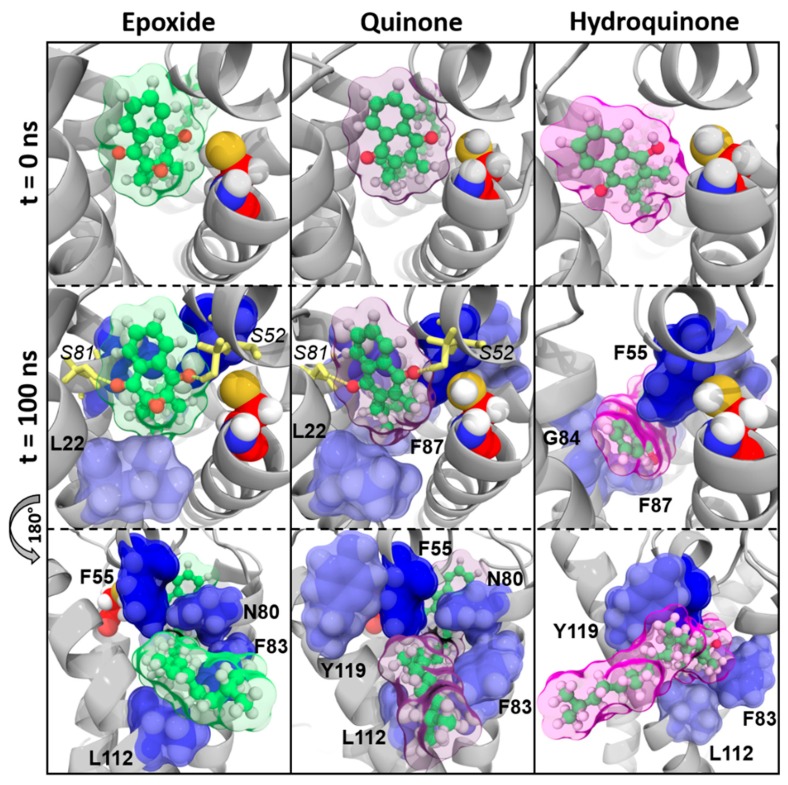
Vitamin K1 interactions with vitamin K epoxide reductase (VKORC1) in molecular dynamics (MD) simulations. Vitamin K1 is shown as green sticks and spheres; the transparent surface is colored as green, violet, and pink for epoxide, quinone, and hydroquinone states of vitamin K1, respectively. The VKORC1 protein structure is depicted as a grey image; the C135 residue is presented as red spheres. Oxygen, nitrogen, sulfur, and hydrogen atoms are colored in red, blue, yellow, and white, respectively. VKORC1 residues involved in hydrogen bonds with vitamin K1 are shown as yellow sticks (hydrogen bonds are depicted as yellow dashes) and numbered in italic characters. VKORC1 residues contributing to vitamin K1–VKORC1 binding free energy are presented as blue spheres and transparent surface (the darker the blue is, the stronger the interaction is) and numbered in bold characters.

We first observed that the location of vitamins K1^E^ and K1^Q^ with respect to the C135 residue was conserved in MD simulations, as shown on *time* = 0 ns and *time* = 100 ns pictures (Figure 3). Two hydrogen bonds carried by S52 and S81 residues retained vitamins K1^E^ and K1^Q^ inside VKORC1. The naphthoquinone head of vitamin K1 was kept close to C135 by F55 and N80 residues, whereas the hydrophobic tail was obviously stabilized by F83, F87, L112, and Y119 residues.

Conversely, vitamin K1^H^ slid away from C135 residue and was close to TM2 at the end of MD simulations. This can be explained by the loss of hydrogen bonds (S52 and S81) and the N80 interaction. Stabilization by F83, F87, G84, L112, and Y119 residues seemed not to be strong enough to keep vitamin K1^H^ close to the catalytic site.

##### Vitamin MK4

**Figure 4:**
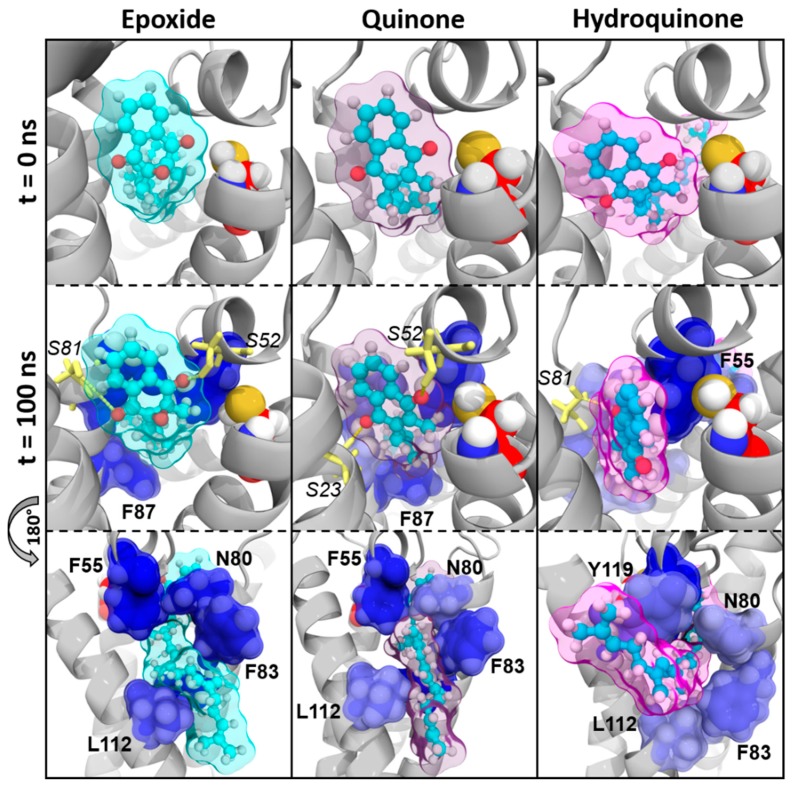
Vitamin MK4 interactions with vitamin K epoxide reductase (VKORC1) in molecular dynamics (MD) simulations. Vitamin MK4 is shown as cyan sticks and spheres; the transparent surface is colored as cyan, violet and pink for epoxide, quinone, and hydroquinone states of vitamin MK4, respectively. The VKORC1 protein structure is depicted as a grey image; the C135 residue is presented as red spheres. Oxygen, nitrogen, sulfur, and hydrogen atoms are colored in red, blue, yellow, and white, respectively. VKORC1 residues involved in hydrogen bonds with vitamin MK4 are shown as yellow sticks (hydrogen bonds are depicted as yellow dashes) and numbered in italic characters. VKORC1 residues contributing to vitamin MK4–VKORC1 binding free energy are presented as blue spheres and a transparent surface (the darker the blue is, the stronger the interaction is) and numbered in bold characters.

Vitamin MK4 exhibited the same binding profile as vitamin K1: vitamins MK4^E^ and MK4^Q^ were close to C135 residue in MD simulations (Figure 4). The naphthoquinone head was kept close to the catalytic site by S52 and S23/S81 residues (hydrogen bonds) on one hand, and by F55 and N80 residues (BFE contribution) on the other hand. The hydrophobic chain was stabilized by F83, F87, and L112 residues.

Nonetheless, vitamin MK4^H^ was also sliding away from VKORC1 catalytic site, with the naphthoquinone head facing TM1 at the end of MD simulations. Stabilization of the hydrophobic tail by F55, N80, F83, L112, and Y119 residues kept vitamin MK4^H^ inside VKORC1, but the loss of the S52 hydrogen bond led to naphthoquinone head displacement with respect to the C135 residue.

##### Vitamin MK7

**Figure 5:**
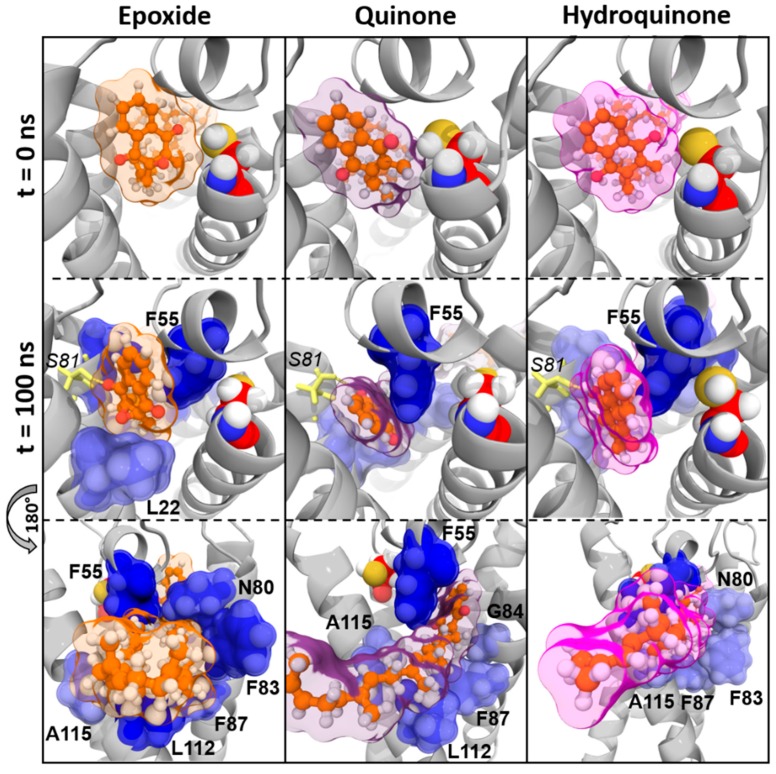
Vitamin MK7 interactions with vitamin K epoxide reductase (VKORC1) in molecular dynamics (MD) simulations. Vitamin MK7 is shown as orange sticks and spheres; the transparent surface is colored as orange, violet, and pink for epoxide, quinone, and hydroquinone states of vitamin MK7, respectively. The VKORC1 protein structure is depicted as a grey image; C135 residue is presented as red spheres. Oxygen, nitrogen, sulfur, and hydrogen atoms are colored in red, blue, yellow and white, respectively. VKORC1 residues involved in hydrogen bonds with vitamin MK7 are shown as yellow sticks (hydrogen bonds are depicted as yellow dashes) and numbered in italic characters. VKORC1 residues contributing to vitamin MK7–VKORC1 binding free energy are presented as blue spheres and a transparent surface (the darker the blue is, the stronger the interaction is) and numbered in bold characters.

Vitamin MK7 binding profile was a little different from vitamins K1 and MK4. Indeed, the naphthoquinone head systematically moved away from C135 residue (Figure 5), induced by the loss of S52 hydrogen bond, even for epoxide and quinone states. However, F55, N80, F83, F87, and L112 residues still contributed to vitamin MK7 stabilization, assisted by the A115 residue. 

##### Vitamin K3

**Figure 6:**
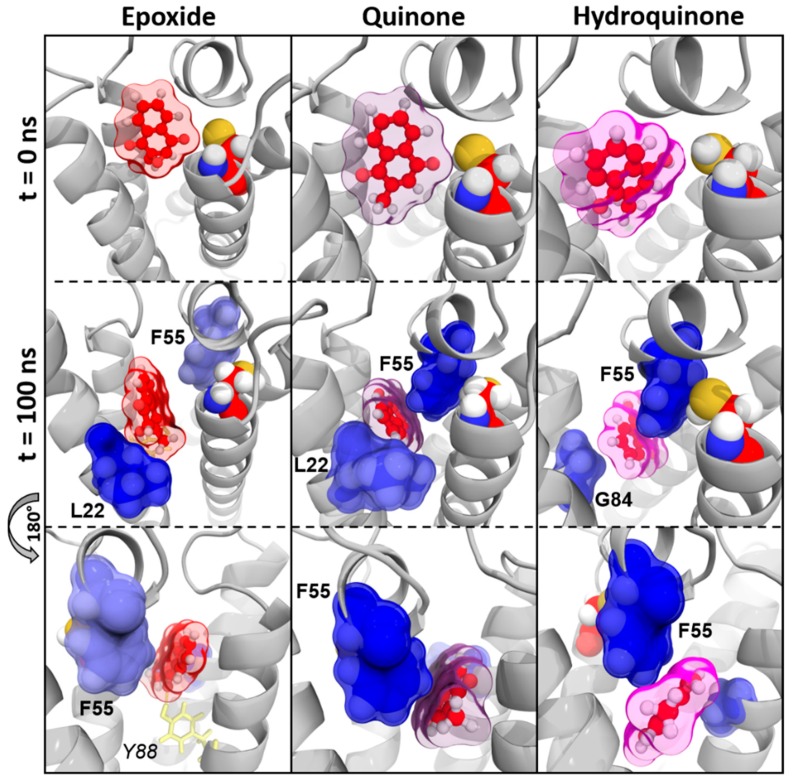
Vitamin K3 interactions with vitamin K epoxide reductase (VKORC1) in molecular dynamics (MD) simulations. Vitamin K3 is shown as red sticks and spheres; the transparent surface is colored as red, violet, and pink for epoxide, quinone, and hydroquinone states of vitamin K3, respectively. The VKORC1 protein structure is depicted as a grey image; the C135 residue is presented as red spheres. Oxygen, nitrogen, sulfur, and hydrogen atoms are colored in red, blue, yellow, and white, respectively. VKORC1 residues involved in hydrogen bonds with vitamin K3 are shown as yellow sticks (hydrogen bonds are depicted as yellow dashes) and numbered in italic characters. VKORC1 residues contributing to vitamin K3–VKORC1 binding free energy are presented as blue spheres and a transparent surface (the darker the blue is, the stronger the interaction is) and numbered in bold characters.

Vitamin K3 was constantly sliding away from C135 residue (regardless of epoxide, quinone or hydroquinone state) and was facing TM2 at the end of MD simulations (Figure 6). Only one hydrogen bond was observed for vitamin K3^E^, involving the Y88 residue. The F55 residue was always contributing to vitamin K3 interactions with VKORC1. Either L22 or G84 were also involved in vitamin K3 binding, depending on the epoxide, quinone, or hydroquinone state.

### 3.3. Membrane Effect of Vitamins K Binding to VKORC1

Finally, we oriented our latest analysis towards vitamin K–membrane interactions. Indeed, the main difference between vitamin MK7 and vitamins K1/MK4 was the hydrophobic tail, and this tail was likely to interact with the membrane.

**Figure 7:**
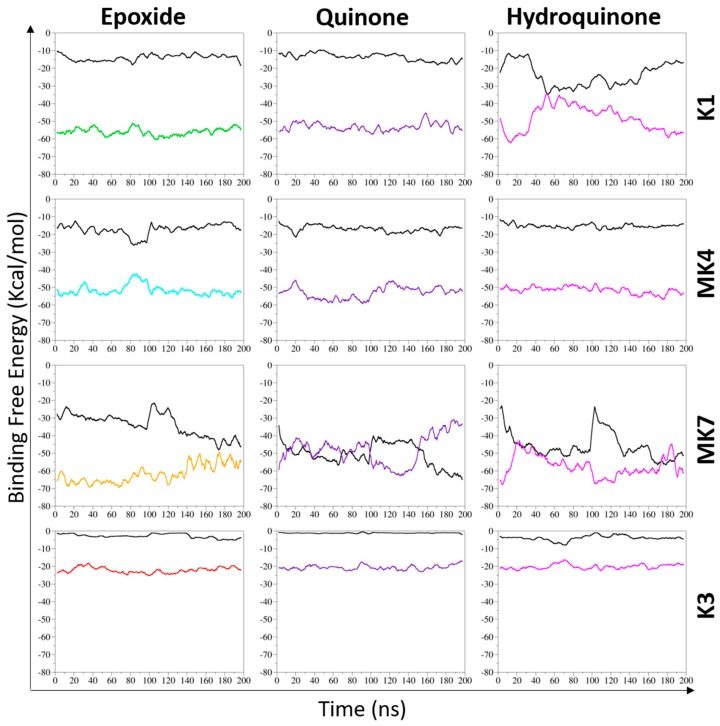
Binding free energy of vitamins K (K1, MK4, MK7, and K3 in their epoxide, quinone, and hydroquinone states) towards vitamin K epoxide reductase (VKORC1) and membrane in molecular dynamics (MD) simulations. The binding free energy (BFE) between vitamin K and membrane is shown as black line. The BFEs between the epoxide state of vitamin K and VKORC1 are shown as green, cyan, orange, and red lines for vitamin K1, MK4, MK7, and K3, respectively. The BFEs of quinone and hydroquinone states of vitamins K towards VKORC1 are presented as violet and pink lines, respectively. Two 100-ns MD simulations were performed on each vitamin K–VKORC1 complex, then concatenated to be considered as one 200-ns MD simulation.

BFE calculation was thus applied to vitamins K towards membrane, to investigate potential differences between vitamins K affinity regarding the lipid bilayer. The vitamin K–membrane overall BFE was finally compared to the vitamin K–VKORC1 BFE (Figure 7).

Distinct BFE profiles were observed depending on the different studied vitamins K, but one point remained constant: vitamins K–membrane and vitamin K–VKORC1 profiles appeared to be symmetrical. When the vitamin K–membrane BFE increased, vitamin K–VKORC1 decreased, and vice versa. This suggested a balance between vitamins K affinity towards the membrane and VKORC1.

All states of vitamin MK4 exhibited stable values (despite the naphthoquinone head displacement regarding vitamin MK4^H^) as well as vitamins K1^E^ and K1^Q^: vitamin K–VKORC1 BFE values remained between −45 and −60 kcal/mol in MD simulations, and the vitamin K–membrane BFE kept constant around −15 kcal/mol. However, the vitamin K1^H^ profile was different with both BFEs, converging to a −35 kcal/mol value at 50 ns. These results agreed with structural observations: vitamin MK4 and vitamins K1^E^/K1^Q^ remained inside the VKORC1 enzyme and poorly interacted with the membrane, whereas vitamin K1^H^ was sliding away from the catalytic site, allowing contacts between the hydrophobic tail and the membrane (Figure 3 and Figure 4).

Vitamin K3–VKORC1 BFE kept constant between −15 and −25 kcal/mol, and the vitamin K3–membrane remained around zero, as expected considering the absence of hydrophobic tail.

Vitamin MK7 revealed the most striking BFE profile: affinity towards VKORC1 and the membrane presented the highest fluctuations values in MD simulations, constantly converged, and even crossed each other for vitamins MK7^Q^ and MK7^H^. The convergence value was between −45 and −50 kcal/mol. This finding revealed a competition between VKORC1 and membrane affinities towards vitamin MK7. Membrane binding was stronger than VKORC1 during 50 ns and 100 ns for vitamins MK7^Q^ and MK7^H^, respectively.

### 3.4. VKORC1 Activity Towards Vitamins K1, MK4, MK7, and K3

**Figure 8:**
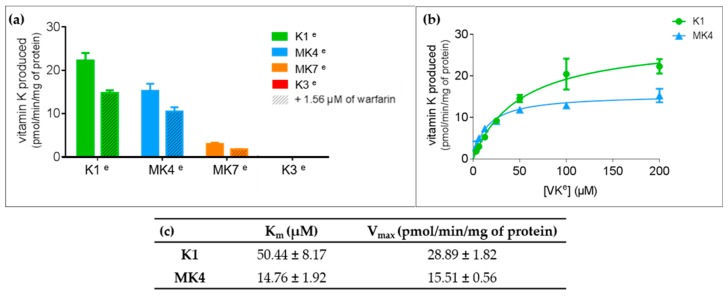
Vitamin K epoxide reductase (VKOR) activity assays and kinetics. (**a**) Specific vitamin K epoxide reductase activity towards vitamins K1^E^, MK4^E^, MK7^E^, or K3^E^. VKOR activity was evaluated in the presence of 100 µM of vitamins K^E^ in the presence of 0 or 1.56 µM warfarin. (**b**) VKOR activity versus vitamins K1^E^ or MK4^E^ incubated with microsomal proteins expressing membrane wild-type human VKORC1. Each data point represents the mean ± standard deviation of three determinations in duplicates. Experimental results were fitted by nonlinear regression using the noncompetitive inhibition model. (**c**) Apparent kinetic parameters towards vitamins K1^E^ or MK4^E^. To determine the VKOR activity, standard reactions were performed in 200 mM 4-(2-hydroxyethyl)-1-piperazineethanesulfonic acid (HEPES) buffer, pH 7.4, containing 150 mM KCl and 1.5 g·L^−1^ microsomal proteins expressing membrane wild-type VKORC1. Each data point represents the mean ± standard deviation of three individual determinations in duplicates.

In vitro ability of VKORC1 to reduce vitamins K1^E^, MK4^E^, MK7^E^, and K3^E^ at different concentrations (i.e., 10 or 100 µM) was evaluated using recombinant human VKORC1 expressed in membranes of *Pichia pastoris*, (Figure 8 and Figure A1). Human VKORC1 was able to reduce vitamins K1^E^ and MK4^E^, with an activity of 23 and 15 pmol/min/mg of protein in the presence of 100 µM of substrate. Addition of 1.56 µM of warfarin inhibited almost 40% of this activity, whether the substrate was vitamin K1^E^ or MK4^E^. Kinetic parameters towards vitamins K1^E^ or MK4^E^ were determined and are presented in Figure 8c. Catalytic efficiencies (i.e., Vmax/Km) towards vitamins K1^E^ and MK4^E^ were respectively 0.57 and 1.05 nL/min/mg of protein. In the presence of 100 µM of MK7^E^, VKOR activity was very limited (~10 times lower than with vitamin K1^E^) preventing determination of kinetic parameters. No VKOR activity was observed in the presence of 100 µM of vitamin K3^E^. Based on the experimental conditions and LC-MS analysis method used, a detection threshold of 2 pmol of vitamin K3^Q^ produced by 1 mg of VKORC1 per minute is ensured. Thus, this absence of signal indicated a VKOR activity towards vitamin K3^E^ below 2 pmol/min/mg of protein, considered as null.

## 4. Discussion

The main objective of the present work was the investigation of VKORC1 preferential substrates among vitamins phylloquinone (K1), menaquinones (K2), and menadione (K3). Indeed, VKOR activity is of main importance in all tissues: in the liver to support blood coagulation and in extrahepatic tissues for the activation of osteocalcin and matrix Gla protein (MGP), VKDPs are involved in energy metabolism and calcification processes, respectively. However, vitamin K tissue distribution is heterogenous, with vitamin K1 mainly present in the liver and vitamin K2 found in high levels in extrahepatic tissues [23]. This raised the issue of the ability VKORC1 enzyme to recycle vitamins K1 and K2 in the same way to ensure VKDP activation in the different tissues, depending on their vitamin K status. Vitamin K3 has been described as unable to activate GGCX or initiate the coagulation mechanism [27,28], but the causes remain unclear: Is it due to menadione pharmacodynamics or is it induced by the inability of VKORC1 to bind (and thus recycle) vitamin K3? Structural investigation of vitamin K3~VKORC1 interactions, validated by in vitro assay, disclosed the reasons of this inactivity

Here we report a molecular modeling characterization of the ability of VKORC1 to bind vitamins K1, K2 (MK4 and MK7), and K3. We also considered epoxide (^E^), quinone (^Q^), and hydroquinone (^H^) states of each vitamin K, to obtain an overview of their binding to VKORC1. VKORC1 activity towards vitamins K1^E^, MK4^E^, MK7^E^ and K3^E^ was finally assessed by in vitro assays.

Molecular modeling studies were performed using our previously developed VKORC1 3D model, including a C51–C132 disulfide bridge, C135 being the catalytic residue. This conformation has been experimentally and in silico proved to be the active conformation of the VKORC1 enzyme, able to bind vitamin K1^E^ [30,31,32], regardless of the differences between the structural models established by these research teams. 

First of all, vitamins K1, MK4, MK7, and K3 in their epoxide, quinone, and hydroquinone states were docked on our VKORC1 structural model (Figure A2). All vitamin K forms bound the same site, the naphthoquinone head being close to C135 residue and facing transmembrane helices (TMs) 1 and 4. The docking results were then used to perform molecular dynamics (MD) simulations, 100 ns in replica, on each vitamin K–VKORC1 complex.

Our first analysis was based on a previously suggested reduction mechanism of vitamin K^E^ to vitamin K^Q^, involving a nucleophilic attack of vitamin K^E^ by C135 residue of VKORC1 [21]. Thus, we first measured distance between the carbon-2 atom of vitamin K^E^ and the C135 α-carbon atom in MD simulations (Figure 1 and Figure A2). The BFE was then calculated for each type of vitamin K–VKORC1 to estimate relative affinities of vitamin K towards VKORC1 (Table 1), following by VKORC1 residue contribution estimation (Figure 2). Amino acid contribution was then considered on 3D structure and coupled with hydrogen bond characterization to obtain a structural overview of vitamin K binding (Figure 3, Figure 4, Figure 5 and Figure 6). Membrane interactions towards the different K vitamins was further investigated through BFE calculations (Figure 7) to evaluate vitamin K–membrane affinities and define the membrane effect on vitamin K binding to VKORC1. Finally, we assessed in vitro VKORC1 reductase activity towards vitamins K1, MK4, MK7, and K3 (Figure 8).

### 4.1. Vitamin K1

Vitamins K1^E^ and K1^Q^ appeared to remain close to the C135 residue in MD simulations (Figure 1) and revealed equivalent affinities based on BFE calculations (Table 1). The VKORC1 amino acid contribution indicated the main importance of F55, N80, F83, F87, and L112 residues for vitamin K1 stabilization (Figure 2). F55 and N80 appeared as keeping the naphthoquinone head close to the C135 residue, whereas F83, F87, and L112 residues were mainly involved in hydrophobic tail stabilization (Figure 3); moreover, two hydrogen bonds (S52 and S81 residues) were identified in vitamin K1^E^ and K1^Q^ binding.

However, vitamin K1^H^ moved away from C135 in MD simulations and BFE indicated lower affinity and higher standard deviation (SD) values compared to vitamin K1^E^ and K1^Q^. These differences might be explained by the loss of S52 hydrogen bond and N80 residue stabilization, leading to the sliding motion of vitamin K^H^ away from C135 (Figure 3). 

BFE calculation focused on the vitamin K1–membrane interactions revealed poor interactions between vitamins K1^E^/K1^Q^ and membrane (Figure 7). Nevertheless, vitamin K1^H^ showed increasing affinity towards the membrane in MD simulations, induced by its sliding outside of VKORC1 and magnifying hydrophobic tail interactions with the membrane.

Finally, in vitro assays indicated VKORC1 ability to reduce vitamin K1^E^ (Figure 8). Taken all together, our results were consistent with previous publications showing the ability of VKORC1 to reduce vitamin K^E^ to K^Q^ and vitamin K^Q^ to K^H^ [14,15], and characterizing vitamin K1^E^ binding to VKORC1. Structural investigation performed here provided accurate insights into VKORC1 features responsible for enzymatic activity towards vitamin K1. Indeed, vitamins K1^E^ and K1^Q^ tightly bound to VKORC1: S52 and S81 residues were involved in hydrogen bonds retaining vitamin K inside the enzyme, F55 and N80 residues kept the naphthoquinone head close to C135, and F83, F87, and L112 stabilized the hydrophobic tail. The F55 residue has previously been identified as the key residue for vitamin K1^E^ binding [30,31], but the S52 hydrogen bond also appeared as crucial, since its loss led to the release of vitamin K1^H^ outside from the catalytic site. These structural features were further used to predict VKORC1 activity towards vitamins MK4, MK7 and K3.

### 4.2. Vitamin MK4

The vitamin MK4 binding profile appeared to be similar to vitamin K1: vitamins MK4^E^ and MK4^Q^ remained close to C135 in MD simulations (Figure 1) and were stabilized by S52 and S81 (S23 for vitamin MK4^Q^) hydrogen bonds (Figure 4). The F55 and N80 residues retained the naphthoquinone head close to C135, and F83, F87, and L112 residues interacted with the hydrophobic tail. Concerning vitamin MK4^H^, the S52 hydrogen bonds was also lost, inducing a slight rotation of the naphthoquinone head. However, BFEs calculated towards VKORC1 were all close and showed low SD values for all vitamin MK4 states (Table 1), even vitamin MK4^H^. Membrane BFE also remained stable in MD simulations (Figure 7). These observations can be explained by the hydrophobic tail, showing less flexibility compared to vitamin K1 and stabilized by the Y119 residue (not found in vitamin K1 binding), preventing vitamin MK4^H^ from being totally released out from the active site. Considering these results, VKORC1 was expected to show similar activity towards vitamin MK4^E^, as with vitamin K1^E^. This hypothesis was attested by in vitro assays, revealing an equivalent VKOR activity towards vitamins K1^E^ and MK4^E^.

### 4.3. Vitamin MK7

Vitamin MK7 investigation revealed a slightly different binding profile: vitamin MK7^Q^ moved away from C135 residue in MD simulations (Figure 1 and Figure 5), calculated BFE towards VKORC1 indicated higher affinity but greater SD values (Table 1), highlighting the shakiness of vitamin MK7 binding. Moreover, the S52 hydrogen bond was systematically lost, inducing a naphthoquinone head rotation or release for vitamin MK7^Q^ (Figure 5). A115 also appeared as a new residue stabilizing the hydrophobic tail. However the most striking observation concerned BFE calculated between the membrane and vitamin MK7: affinity towards the membrane constantly increased over MD simulations, converging with VKORC1 affinity and even becoming greater (Figure 7). These results explained the instability of vitamin MK7 binding and the loss of S52 hydrogen bond: membrane interactions were stronger than VKORC1 affinity, leading to vitamin MK7 binding failure. Based on these molecular modeling features, VKORC1 activity towards vitamin MK7^E^ was expected to be much lower than towards vitamins K1^E^ and MK4^E^. In vitro characterization indicated a weak activity, 10 times lower than vitamin K1^E^, consistent with our in silico prediction.

### 4.4. Vitamin K3

Vitamin K3 presented the most simple binding profile: all states appeared to move away from the active site in MD simulations (Figure 1 and Figure 6), and the calculated BFE indicated an affinity at least half than for other vitamins K (Table 1), structural examination showed no hydrogen bonds (except with the Y88 residue for vitamin K3^E^) and only L22, F55, and G84 residues contributed to the BFE (Figure 6), because of the absence of hydrophobic tail. In addition, membrane BFE calculation pointed out no interactions between vitamin K3 and the lipid bilayer (Figure 7). Our molecular modeling results thus tended to prove that VKORC1 is unable to bind vitamin K3, because of the absence of hydrophobic tail. In vitro VKORC1-specific activity towards vitamin K3^E^ was finally investigated, and indicated no activity. This absence of signal explained the absence of menadione biological activity previously reported [27,28], due to VKORC1 inability to reduce vitamin K3.

Taken together, these results generated and overall and accurate structural characterization of vitamins K binding to VKORC1, consistent with in vitro observed activity that validated our in silico model. To be reduced by the enzyme, vitamin K is stabilized by two hydrogen bonds (S52 and S81 or S23, S52 being crucial) which is not possible for the hydroquinone state. The naphthoquinone head is kept close to C135 residue by F55 and N80 residues, whereas the hydrophobic tail is mainly stabilized by F83, F87 and L112 residues.

**Figure A3:**
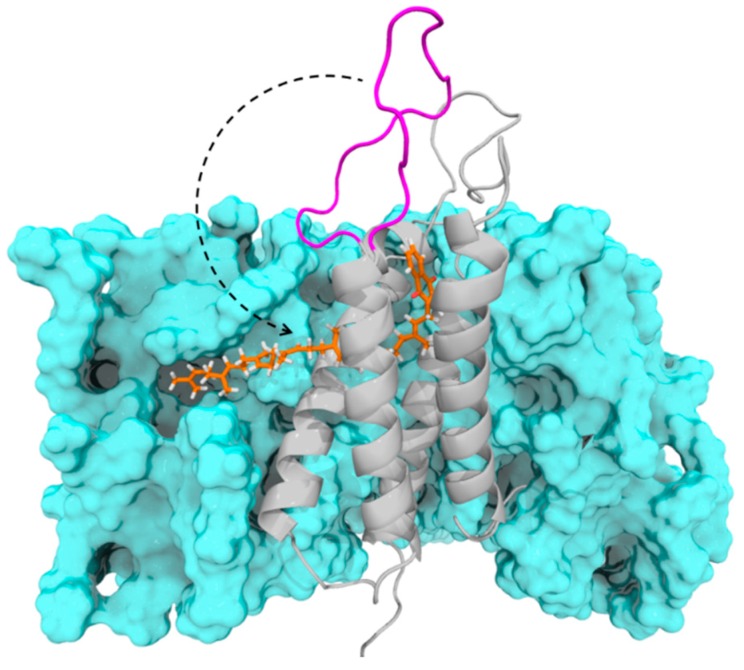
Vitamin MK7 hydrophobic tail interactions with the membrane. The three-dimensional vitamin K epoxide reductase (VKORC1) structure is shown as a grey image, with vitamin MK7^E^ as orange sticks. The membrane is depicted as a light blue surface. The black dashed arrow indicates the location of the magenta loop part which is embedded into the membrane with respect to the VKORC1 structural models developed by Oldenburg [30] and Li [39].

We also evidenced the major role of the vitamin K hydrophobic tail: its absence prevented structural stabilization inside VKORC1 and such VKOR activity (vitamin K3), whereas a long-sized hydrophobic chain induced a membrane effect on vitamins K binding to VKORC1, depending on the vitamins K hydrophobic tail size: Membrane affinity appeared to increase with the hydrophobic tail size, tearing vitamin MK7 apart from the active site regardless the epoxide, quinone, or hydroquinone state. This last observation is consistent with a previous study showing a decrease in the biological activity of vitamin K as the hydrophobic tail size increases [26]. However, this key point concerning vitamin K–membrane interactions could only be handled using our structural model, which would not have been the case regarding the two other structural models developed by Li [30] and Oldenburg [31]. Indeed, our model considers the VKORC1 loop as totally located into the endoplasmic reticulum lumen (Figure A3), whereas Li and Oldenburg 3D models indicated a part of this loop as inside the membrane [30,31], closing the VKORC1 catalytic pocket and preventing the vitamins K hydrophobic tail from interacting with the membrane.

As a conclusion, we showed the ability of VKORC1 to reduce vitamins K1 and MK4, but not MK7 and K3. These findings explained VKORC1 ability to support VKPD activation in the liver (mainly containing phylloquinone) and in extrahepatic tissues (in which menaquinones constitute the most of vitamin K stocks). Membrane interactions with the vitamins K hydrophobic tail raised a new questions: Are long-sized hydrophobic tail menaquinones able to act as GGCX cofactors? Are they eliminated (because they are unrecycled by VKORC1) or stocked and then converted to MK (as menadione might be a precursor of menaquinones) [29]? What would be the ideal hydrophobic tail size to obtain a better compromise between VKORC1 stabilization and membrane affinity? One further study could handle this point, focusing on the number of isoprene units from which the affinity towards membranes becomes higher than towards VKORC1. The activity of VKOR towards menaquinones might be evaluated in vitro and in silico, starting from MK1 to MK9. Such an investigation would first check the hydrophobic chain size from which vitamin K can be structurally stabilized (and thus recycled) by VKORC1, and would also shed light on specific menaquinones reduced by VKORC1. Another molecular modeling investigation might be performed on the direct interactions of menaquinones with the membrane without VKORC1, to study the probable retention of some long-tailed vitamins K2 inside the membrane. Some other questions still remain unanswered: VKORC1 seemed to reduce both vitamins K^E^ and K^Q^, but is this mechanism sequential, with vitamin K remaining inside VKORC1? Or does vitamin K need to go outside from the enzyme then come back, while VKORC1 regenerates its reducing power? Does the C51–C135 disulfide bridge constitute the most adequate conformation to bind vitamin K^H^? The C51–C135 disulfide bridge has been shown to be the primary VKORC1 conformation [30], but further analysis should be carried out to address these questions, considering other VKORC1 conformations and using MD simulations involving quantic mechanics calculations to accurately describe the enzymatic reaction catalyzed by VKORC1.

Nevertheless, this article might constitute a precious starting point for future vitamin K-based therapies, since it gives an overview of the different forms of vitamin K interactions with VKORC1. It also highlighted the importance of membrane consideration into affinity determination, a fact that could also be of great importance for VKORC1 inhibitor development.

## 5. Conclusions

The molecular modeling study performed here evidenced vitamin K epoxide reductase (VKORC1) ability to bind both vitamin K1 and vitamin MK4 in their epoxide and quinone state, but not in the hydroquinone state. Moreover, vitamins MK7 and K3 were not structurally stabilized inside VKORC1, regardless the epoxide, quinone or hydroquinone state. These results were assessed by *in vitro* activity assays, that revealed VKOR activity towards vitamins K1 and MK4 epoxide, but not regarding vitamins MK7 or K3 epoxide. Structural investigation of vitamins K–VKORC1 complexes indicated seven VKORC1 residues essential for vitamin K binding: S52 and S81 residues were involved in hydrogen bonds, F55 and N80 residues kept the vitamin K naphthoquinone head close to the catalytic site (C135 residue), and F83, F87 and L112 residues stabilized the hydrophobic tail. Finally, this tail size appeared to be crucial, since an absence of hydrophobic tail prevented vitamin K binding (vitamin K3) to VKORC1, whereas a long-sized tail led to a membrane effect pulling vitamin K outside the enzyme (vitamin MK7). Taken together, these results explained VKORC1 ability to support vitamin K reduction in the liver and extrahepatic tissues, depending on their vitamin K status.

## Figures and Tables

**Table 1:** Binding free energy (BFE) of vitamin K–VKORC1 complexes (kcal/mol).

	Epoxide	Quinone	Hydroquinone
K1	−55.91 ± 3.73	−53.22 ± 3.85	−48.48 ± 7.79
MK4	−51.56 ± 4.35	−52.99 ± 4.49	−51.49 ± 3.35
MK7	−61.67 ± 7.24	−47.96 ± 9.95	−57.42 ± 7.49
K3	−22.19 ± 2.37	−20.65 ± 2.36	−20.19 ± 2.15
